# Special Issue on Intelligent Systems in Sensor Networks and Internet of Things

**DOI:** 10.3390/s20113182

**Published:** 2020-06-03

**Authors:** Chang Choi, Gianni D’Angelo, Francesco Palmieri

**Affiliations:** 1 Computer Engineering, Gachon University, Seongnam-si 13120, Gyeonggi-do, Korea; changchoi@gachon.ac.kr; 2 Department of Computer Science, University of Salerno, 84084 Fisciano (SA), Italy; giadangelo@unisa.it

## Abstract

This Special Issue aims at collecting several original state-of-the-art research experiences in the area of intelligent applications in the IoT and Sensor networks environment, by analyzing several open issues and perspectives associated with such scenarios, in order to explore novel potentialities and solutions and face with the emerging challenges.

## 1. Introduction

In the last decade, the rapid developments in hardware, software, and communication technologies have facilitated the spread of sensors, actuators and heterogeneous devices connected via the Internet (referred to as the Internet of Things, in brief IoT, which collect and exchange huge amounts of data to offer a new class of advanced services characterized by being available anywhere, at any time and to anyone. Nevertheless, without intelligence, the IoT systems and, in general, sensor networks can act only as ordinary information systems based on predefined rules. On the contrary, adding artificial intelligence (AI) to the mix may allow services to be provided according to users’ habits, activities, and real-world contexts. Combining AI with the IoT opens the world to unlimited technological potential.

The intelligent processing of IoT data, and the building of intelligent systems able to make autonomous decisions, are the keys to developing smart IoT applications and services. The combination of different scientific fields that use data mining, machine learning, and other AI techniques, has proven to be effective in exploring and handling the huge amount of data generated by IoT systems and sensor networks. In addition, other intelligences based on heuristic approaches, such as simulated annealing, genetic algorithms, evolutionary algorithms, ant colony optimization, and particle swarm optimization, have also proven to be effective in making the IoT systems and sensor networks aware of events and contexts, especially when dealing with large amounts of incomplete or inconsistent data.

Accordingly, the goal of this Special Issue has been featuring the latest advances and directions in this amazing evolution process by exploring the potential of new architectures, protocols and services, as well as investigating novel methodologies, theories, systems, and applications for the introduction of intelligence in IoT systems and sensor networks.

We are sure that the experiences presented in this Special Issue may significantly contribute to the work and studies conducted by academic researchers, industry professionals, students, and everyone interested in this subject wanting to extend their knowledge about the above scenarios.

## 2. Special Issue Contents

This Special Issue is composed of 13 contributions, carefully selected according to their subject and accepted based on merit contents. These works cover a variety of topics, essentially related to introduction of intelligence in the IoT environment. 

The paper [1] presents a new data reduction solution that, unlike the traditional schemes, such as deduplication and compression, makes use of the identification of relevant data and correlations among heterogeneous datasets by achieving significant results in terms of data volume reduction. 

The paper [2] derives a novel and exact closed-form expression, more accurate than the existing ones, for energy detection in terms of Meijer’s G-function over α-μ generalized fading channels that can be used in various areas of wireless communications, especially in the wireless sensors and the cognitive radio networks ones.

The paper [3] proposes a cooperative traffic signal control approach based on Deep Q-Network extensions for fast and stable learning, combined with traffic flow prediction to mitigate traffic congestion at multiple intersections.

The paper [4] presents a novel feature point matching approach based on distinct wavelength phase congruency and log-Gabor filters for infrared and visible images, that exhibits superior performance compared to other state-of-the-art approaches. 

The paper [5] proposes a novel adaptation of the multi-group quasi-affine transformation evolutionary algorithm for global optimization to be used for improving node localization accuracy in wireless sensor networks.

The paper [6] presents a novel seasonal time series forecasting algorithm based on the direct and inverse F^1^-transform aiming at improving the performance of the TSSF algorithm, a seasonal time series forecasting method based on direct and inverse F-transform to be used for effectively analysing data gathered from weather stations.

The paper [7] proposes an intelligent rapid adaptive offloading algorithm is proposed for time-varying IoT systems based on the mobile edge computing paradigm. Such algorithm by leveraging a learning-based framework, is able to continually derive adaptive strategies combining offloading decision making with radio resource slicing. 

The paper [8] introduces a low-power distributed data flow anomaly-monitoring model for detecting anomalies in distributed data flows in industrial IoT applications. Such model is able to mitigate the communication overhead by integrating multiple objects in a single complete set, making full use of the relationship between objects. 

The paper [9] presents a novel framework for general neighbour discovery protocols in wireless sensor networks, able to greatly increase the discovery rate and extend the lifetime of sensor nodes.

The paper [10] proposes a new algorithm leveraging a triple filters mixed and Unscented Kalman Filter voting algorithm based on Fuzzy-C-Means to mitigate non-line-of-sight errors and enhance the accuracy of localization in mixed line-of-sight and non-line-of-sight Wireless Sensor Network environments.

The paper [11] presents an energy-efficient and delay tolerant routing scheme that divides the whole network into clusters and elects a head for each cluster by using a weighted sum approach, by not only considering the residual energy of the neighbour nodes but also minimizing the intra-cluster communication cost of a cluster. 

The paper [12] performs a comparative analysis of deep models for convolutional neural network in biometrics using scalogram of electrocardiograms.

The paper [13] discusses the development and application of AI-DTS, a driver training system designed for providing real-time mentoring or post-assessment feedback in driver training, predicting the appropriate moment to rotate the steering wheel in reverse parking. 

Finally, the paper [14] illustrates a framework for a temperature sensor using wireless communication with great potential in facilitating wireless sensing technology in monitoring structural fires. 
